# Calibration of Dual-Channel Raman Spectrometer via Optical Frequency Comb

**DOI:** 10.3390/s24041217

**Published:** 2024-02-14

**Authors:** Shengyujie Lv, Xiaoping Lou, Qiaona Gai, Taotao Mu

**Affiliations:** School of Instrument Science and Opto-Electronics Engineering, Beijing Information Science and Technology University, Beijing 100192, China

**Keywords:** calibration of Raman spectrometer, wide spectrum, optical frequency comb, dual-channel beam splitting structure, dual-band calibration, polynomial fitting

## Abstract

The portable Raman spectrometer boasts portability, rapid analysis, and high flexibility. It stands as a crucial and powerful technical tool for analyzing the chemical composition of samples, whether biological or non-biological, across diverse fields. To improve the resolution of grating spectrometers and ensure a wide spectral range, many spectrometer systems have been designed with double-grating structures. However, the impact of external forces, such as installation deviations and inevitable collisions, may cause differences between the actual state of the internal spectrometer components and their theoretical values. Therefore, spectrometers must be calibrated to establish the relationship between the wavelength and the pixel positions. The characteristic peaks of commonly used calibration substances are primarily distributed in the 200–2000 ${\mathrm{cm}}^{-1}$ range. The distribution of characteristic peaks in other wavenumber ranges is sparse, especially for spectrometers with double-channel spectral structures and wide spectral ranges. This uneven distribution of spectral peaks generates significant errors in the polynomial fitting results used to calibrate spectrometers. Therefore, to satisfy the calibration requirements of a dual-channel portable Raman spectrometer with a wide spectral range, this study designed a calibration method based on an optical frequency comb, which generates dense and uniform comb-like spectral signals at equal intervals. The method was verified experimentally and compared to the traditional calibration method of using a mercury–argon lamp. The results showed that the error bandwidth of the calibration results of the proposed method was significantly smaller than that of the mercury–argon lamp method, thus demonstrating a substantial improvement in the calibration accuracy.

## 1. Introduction

Raman measurement can give the vibrational spectrum of the analyte, which can be treated as its “fingerprint”, allowing easy interpretation and identification. Raman spectrometers are used in the fields of chemistry [1], biomedicine [2], geology [3], food safety [4], and public safety [5] because of their rapid on-site and non-damage measurements, ability to measure aqueous solutions, clear and sharp Raman characteristic peaks, and ease of analysis. They have been developed rapidly and are an important modern analytical technology with a broad range of prospects [6].

Before a spectrometer can be used, it must be calibrated to establish the relationship between the wavelength and the pixel positions [7]. Calibration accuracy is a key indicator of a spectrometer’s performance. At present, a widely used calibration method in Raman spectroscopy is to collect the spectrum of a standard light source or standard substance, obtain data corresponding to the pixels and wavelengths [8], use the least squares method to perform polynomial fitting on this group of data points, establish the formula for the polynomial relationship between the wavelength and the corresponding pixel positions, and then use this formula to map all pixel positions and obtain the corresponding wavelengths [9]. Standard light sources commonly used in the calibration process include mercury–argon lamps [10], neon lamps, holmium oxide solutions [11], holmium glass, and other standard substances with characteristic absorption peaks. The most commonly used standard light source is the mercury–argon lamp. However, with the development of microspectrometers, the detection range and resolution of spectrometers have gradually increased, and many spectrometer systems featuring dual-channel splitting and dual-band detection have been developed. If a spectrometer is calibrated only by the simple method mentioned above, fewer calibration data are obtained in a given spectral range because the characteristic peaks are unevenly distributed. This significantly affects the calibration accuracy because the polynomial fitting used in the calibration forcibly establishes a mapping between the wavelength and the pixel positions, which decreases its accuracy at pixel positions far away from the standard characteristic wavelength. As a result, in areas with few data points, the calibration wavelength is stretched to that of areas in which data points are concentrated, causing the wavelength deviation to increase [12]. Therefore, in a given wavelength range, selecting a higher number of characteristic spectral lines results in a smaller wavelength deviation after calibration.

To address the low precision of the polynomial fitting method, several new wavelength calibration methods have been proposed. For example, Youngquist et al. artificially produced equidistant spectral lines by using a white light interferometer [13]. In addition, Perret et al. used a Fabry–Perot interference filter to produce equidistant spectral lines of equal intensity [14]. Furthermore, Yu et al. used parallel broad-spectrum beams to irradiate double-sided metal-clad planar waveguides and generated a series of comb-like spectra with equal frequency intervals for calibration [15]. Moreover, Wang et al. collected monochromatic light by controlling the scanning of the grating in the monochromator [16]. All of the methods mentioned above can effectively obtain sufficiently uniform calibration data points, but most of them are suitable only for spectrometers with single-grating beam-splitting structures. No systematic calibration method has been proposed for spectrometers with double-channel beam-splitting structures. Moreover, Raman spectrometers use the Raman shift (${\mathrm{cm}}^{-1}$) as the unit of the abscissa. Thus, when using a traditional light source for calibration, the relationship between the wavelength of the light source and the Raman shift of the collected spectrum must be considered, which is inconvenient.

To fulfill the calibration requirements for a dual-channel portable Raman spectrometer with a broad spectral range, this paper introduces a calibration method based on an optical frequency comb. This method generates dense and uniformly spaced spectral signals at equal intervals, ensuring a substantial number of pixel and wavenumber data pairs with a consistent distribution [17]. This addresses the dependency of polynomial fitting on the number and distribution of peak points in the calibration process for spectrometer detection systems with wide spectra and high resolution [18]. Simultaneously, prioritizing user-friendly operation, time efficiency, and high precision, this paper aims to design the entire calibration process using readily available substances and portable instruments. The calibrated system is then employed to analyze various material samples. The discrepancy between the Raman characteristic peak of the material detected by the system and its standard Raman shift is examined, and the error in the calibration results is analyzed. Furthermore, the calibration results are compared with those obtained using the traditional mercury–argon lamp, validating the reliability of the proposed calibration method in this paper.

The rest of this paper is organized as follows: Section 2 describes the structure of the dual-channel portable Raman spectrometer; Section 3 explains the dual-band wavelength calibration method based on the optical frequency comb; Section 4 presents and analyzes the experimental results; and Section 5 contains the conclusions of the study.

## 2. Structure of Dual-Channel Portable Raman Spectrometer

**Figure 1:**
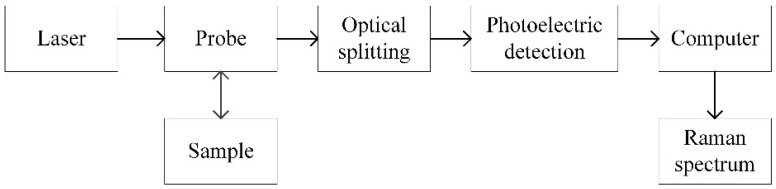
Diagram of the structure of the portable Raman spectrometer used in this study.

The Raman spectrometer used in this study included several parts: an excitation light source, a Raman probe, a light-splitting system, a photoelectric detection system, and a data processing system, as shown in Figure 1. The excitation light source irradiated the sample through the input end of the Raman probe and generated Raman-scattered light via excitation. The signal entering the collection optical path contained the Raman spectrum of the sample to be measured. A notch filter was arranged in the collection optical path to filter out Rayleigh scattering entering the probe. The Raman spectrum of the remaining sample entered the spectroscopic system through the slit. The spectroscopic system dispersed the spectral signal according to the wavelength and transmitted it to the photoelectric detection device, which converted the optical signal into an electrical signal [19]. The signal from the photoelectric detection device was sent to the data processing system to complete the analysis of the Raman spectrum [20].

**Figure 2:**
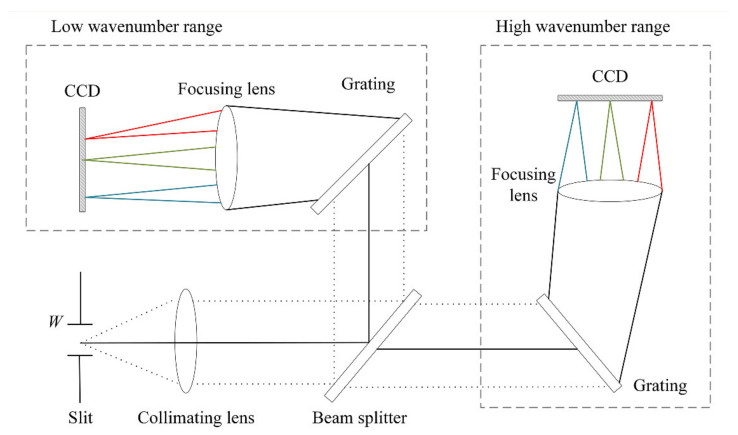
Diagram of the optical path of the beam-splitting system in the Raman spectrometer used in this study.

The volume and resolution of a Raman spectrometer are primarily determined by the optical system, the core of which is the beam-splitting system. The dispersion performance of the light-splitting system determines the resolution of the spectrometer, whereas the structure of the light-splitting path determines the volume of the spectrometer. The spectral structure of the beam-splitting system in the Raman spectrometer used in this study is shown in Figure 2. The system consisted of six parts: an incident slit, collimator, beam splitter, gratings, focusing lenses, and charge-coupled device (CCD) detectors.

Light with a wide wavelength range entered the slit and was collimated into parallel light beams of a certain width. The collimated parallel light then reaches the beam splitter, a semi-transparent mirror that redirects light into transmission and reflection paths according to the wavelength. In this optical path system, light beams with high wavenumbers were transmitted through the beam splitter, and light beams with low wavenumbers were reflected by the beam splitter. In each optical path, the light beams were split by a grating at diffraction angles corresponding to the different wavelengths. Then, they were focused by an imaging mirror and received by a CCD detector. Each CCD pixel received a spectral intensity signal (denoted as *I*) with a specific wavelength, which was recorded. The technical parameters of this spectrometer are shown in Table 1.

In practical applications, the Raman spectrum corresponding to the Raman shift and spectral intensity *I* are analyzed. Because the unit of the Raman shift is the wavenumber (WN), it is necessary to calibrate the spectrometer and obtain the mapping relationship between the wavenumber and each pixel.

## 3. Dual-Band Wavelength Calibration Method Based on Optical Frequency Comb

To create a dual-channel Raman spectrometer, the two detection channels corresponding to the high- and low-wavenumber bands were calibrated. Currently, mercury–argon lamps are the most commonly used wavelength calibration source. However, the spectral peaks of mercury–argon lamps are sparsely distributed over a wide spectral range. During the calibration process, the number of known spectral lines contained in the standard materials should be as large as possible, and the more uniform the distribution, the more accurate the calculation results.

An optical frequency comb (OFC) is a spectrum consisting of a series of uniformly spaced frequency components with coherently stable phase relationships generated by the interaction between an optical signal and a radio frequency signal. OFCs were developed approximately 20 years ago and have become widely used as standard technology. They act as precision optical synthesizers capable of transmitting phase and frequency information from a highly stable reference to hundreds of thousands of frequencies in the optical domain [21]. OFCs have facilitated the development of precise measurement capabilities in both fundamental research and practical applications. The sources used to generate this comb structure include mode-locked lasers [22], optical pump microring resonators [23], narrow-linewidth continuous-wave (CW) laser modulations [24], and optical comb filters [25].

**Figure 3:**
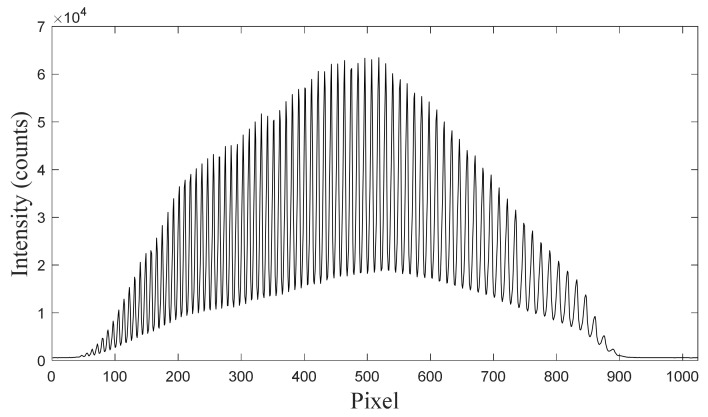
Raman spectrum of the optical frequency comb generated in this study.

In this study, a halogen light source was used to generate stable broad spectral signals, which were converted into optical combs by a waveguide comb filter, as shown in Figure 3. The optical comb filter was implemented using a bimetallic-clad optical waveguide that could convert the broad spectrum of a common lamp into a combined feature spectrum. The structural and material parameters of the waveguide were adjusted such that the light propagating in the waveguide had different transmission characteristics at different wavelengths. As a result, when the broad-spectrum light of an ordinary lamp passed through the waveguide, light beams of different wavelengths were selectively transmitted by specific modes in the waveguide, thus forming a combined characteristic spectrum [15].

**Figure 4:**
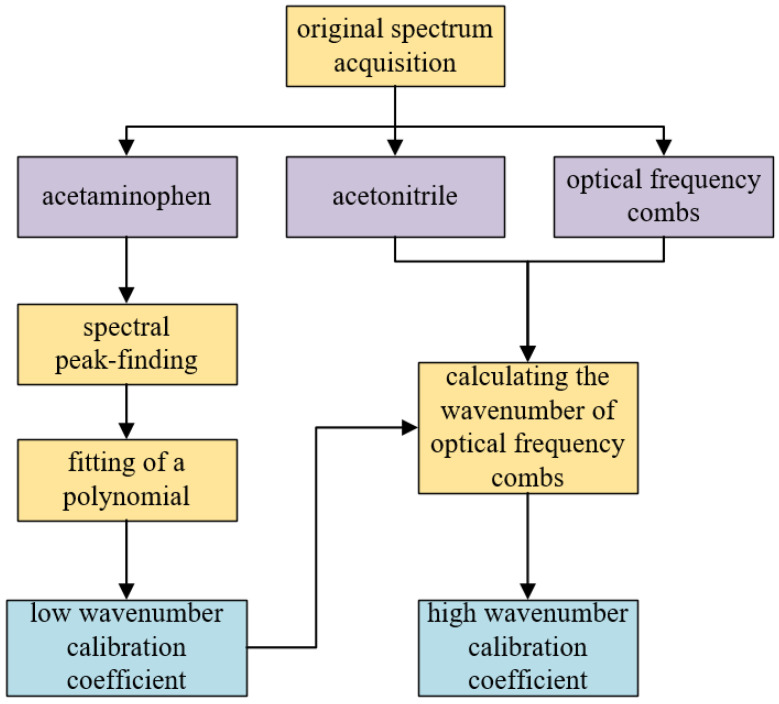
Flow chart of the dual-band calibration method.

Because the Raman shift corresponding to each spectral peak generated by the optical frequency comb was unknown, it was necessary to calculate the wavenumber of each spectral peak. Assuming a fixed optical–mechanical structure, the wavelength of the optical signal collected by each pixel of the CCD was fixed. Therefore, the Raman shift of one or several spectral peaks of the optical frequency comb could be determined from one of the standard material’s characteristic peaks with a known wavenumber. Because OFCs exhibit wavenumber intervals of equal size, only the wavenumber of one spectral peak of the optical frequency comb needs to be determined. To compensate for the dependence of polynomial fitting on the spectral peaks involved in the calibration in the high-wavenumber band, the wavenumber of each spectral peak was calculated in combination with the wavenumber interval of the optical frequency comb. A flowchart of the dual-band calibration method used in this study is presented in Figure 4.

The positions of the spectral peaks affect the accuracy of the wavelength calibration. Therefore, it is necessary to choose an appropriate automatic algorithm to find the optimal position of the pixel corresponding to the peak of each characteristic spectral line. Commonly used spectral peak-finding methods include the centroid method [26], the symmetric zero-area peak-finding method [27], and the Gaussian fitting method [28]. Owing to the physical characteristics of light, the linear spectrum exhibits a Lorentzian shape, and because of the expansion of the CCD spectrometer, the shape of the linear spectrum is usually a symmetric Gaussian function. Hence, the peak value and its corresponding position can be easily obtained with high accuracy via Gaussian fitting of the spectral data [29]. Therefore, in this study, a Gaussian fitting method was used to locate the optimal pixel positions of the spectral peaks.

According to the grating dispersion equation and the geometric relationship between the components of the spectrometer [30], a nonlinear relationship exists between the Raman shift and the CCD pixel position [9]. Because the spectral measurement error directly affects the accuracy of subsequent data analysis, the calibration equation usually takes the form of a high-order polynomial, and the higher the order of the polynomial fitting, the more accurate the fitting result. However, when the order is increased, the improvement in accuracy is not obvious, and if too few data points are involved in the polynomial fitting, overfitting easily occurs. Considering the real-time detection characteristics of a portable Raman spectrometer, the relationship between calibration accuracy and calculation time should be balanced to meet the application requirements. After numerous experiments, a third-order polynomial fit was determined to be the most accurate [31].

**Figure 5:**
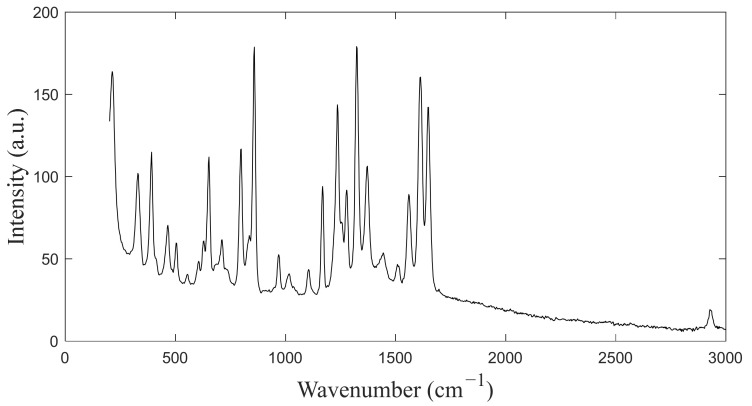
Standard Raman spectrum of acetaminophen.

In the calibration experiment, acetaminophen was selected as the calibration substance for the low-wavenumber band because it has a sufficient number of uniformly distributed Raman reference peaks in the 200–1640 ${\mathrm{cm}}^{-1}$ spectral range. A standard Raman spectrum for acetaminophen is shown in Figure 5. To calculate the calibration coefficient of the low-wavenumber band, the pixel positions of the characteristic peaks were fitted with the standard wavenumber using a cubic polynomial.

The pixel unit of the OFC was mapped to the Raman-shifted wavenumber range using the calibration coefficient of the low-wavenumber band. Using the Gaussian peak-finding algorithm, information such as the wavenumber ${\mathrm{OFC}}_{i-\mathrm{WN}}$ (the abscissa), light intensity *I* (the ordinate), and spectral peak serial number *i* can be obtained. In addition, the total number *N* of spectral peaks of the OFC can be determined according to the maximum serial number, and the wavenumber interval ${\mathrm{OFC}}_{\mathrm{WNspace}}$ can be calculated according to the average value, which is expressed as

(1) $${\mathrm{OFC}}_{\mathrm{WNspace}}=\frac{\sum \left({\mathrm{OFC}}_{\left(i+1\right)-\mathrm{wn}}-{\mathrm{OFC}}_{\left(i+1\right)-\mathrm{wn}}\right)}{N\;-\;1},\;\left(1\;\leq \;i\;<\;N,\;i\;\in Z\right).$$

**Figure 6:**
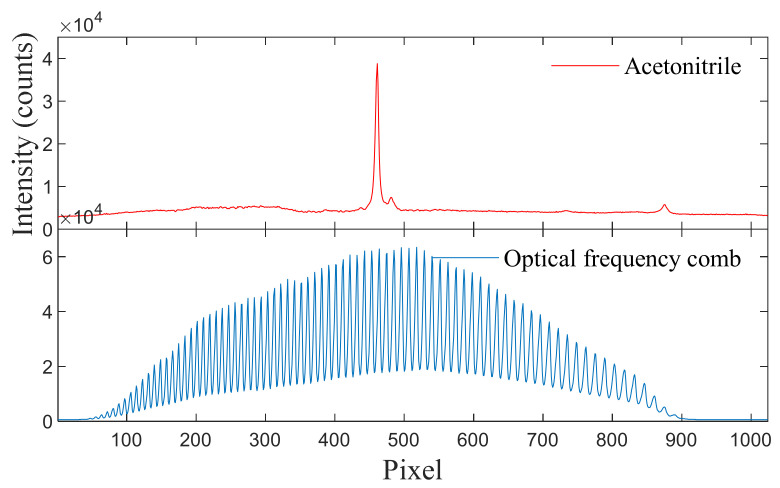
Original spectra of acetonitrile and the optical frequency comb in the high-wavenumber band.

Combined with the value calculated in Equation (1), acetonitrile (ACN) was selected to calibrate the high-wavenumber band to assist in calculating the peak wavenumber of the OFC in that band. Acetonitrile has only one high-resolution and high-intensity Raman peak in the 500–3000$\;{\mathrm{cm}}^{-1}$ spectral range: 2253 ${\mathrm{cm}}^{-1}$ [32]. The measured spectrum of acetonitrile and that of the OFC in the high-wavenumber band are shown in Figure 6.

**Figure 7:**
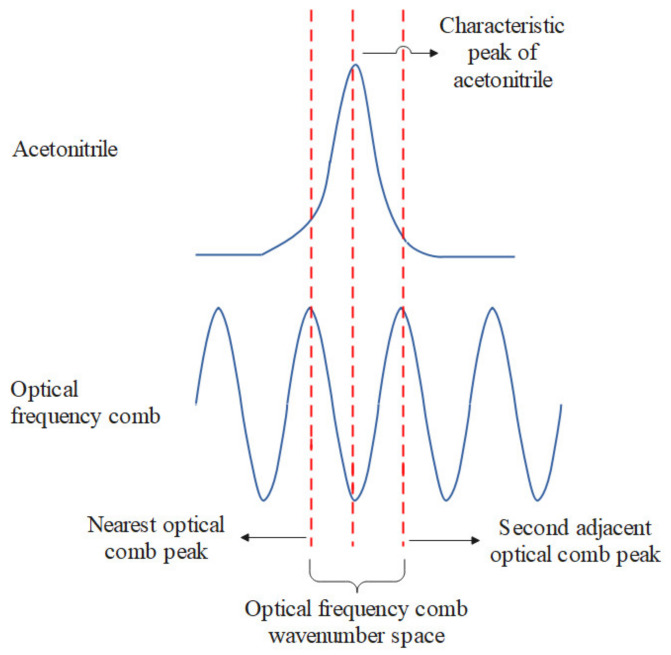
Relative positions of the spectral peaks of acetonitrile and those of the OFC.

Assuming a fixed optical–mechanical structure, the pixel position of the 2253 ${\;\mathrm{cm}}^{-1}$ peak in the acetonitrile spectrogram was equal to the Raman shift of the same pixel position in the OFC spectrogram. The acetonitrile pixel position was denoted as ${\mathrm{ACN}}_{\mathrm{pixel}}$, the OFC peak nearest to that of the acetonitrile was denoted as peak *n*, and its pixel position was denoted as ${\mathrm{OFC}}_{\mathrm{nearest}-\mathrm{Pixel}}$. The value of ${\mathrm{OFC}}_{\mathrm{second}-\mathrm{Pixel}}$ represents the OFC pixel position next to the position of the acetonitrile 2253$\;{\mathrm{cm}}^{-1}$ peak. Assuming that the relationship between the wavenumber distance and the pixel position distance is approximately linear, the relationship between the pixel positions of the acetonitrile peaks and those of the OFC is shown in Figure 7. The wavenumber ${\mathrm{OFC}}_{\mathrm{nearest}-\mathrm{WN}}$ of the OFC peak was calculated using

(2) $${\mathrm{OFC}}_{\mathrm{nearest}-\mathrm{WN}}=2253+\frac{\left({\mathrm{OFC}}_{\mathrm{nearest}-\mathrm{Pixel}}-{\mathrm{ACN}}_{\mathrm{pixel}}\right)}{{(\mathrm{OFC}}_{\mathrm{nearest}-\mathrm{Pixel}}-{\mathrm{OFC}}_{\mathrm{second}-\mathrm{Pixel}})}\;\times \;{\mathrm{OFC}}_{\mathrm{WNspace}}.$$

The wavenumber of the OFC peak *i* was expressed as

(3) $${\mathrm{OFC}}_{i-\mathrm{WN}}={\mathrm{OC}}_{\mathrm{nearest}-\mathrm{WN}}-\left(n-i\right)\;\times \;{\mathrm{OFC}}_{\mathrm{WNspace}}.$$

The wavenumber of each OFC peak was calculated using Equation (3). To derive the calibration result for the high-wavenumber band, the wavenumbers and pixel positions of the OFC spectral peaks were fitted using a polynomial. A Raman spectrum image with a wide spectral range was obtained by splicing the calibrated spectra of the high- and low-wavenumber bands.

## 4. Experimental Results and Analysis

### 4.1. Spectrometer Calibration

**Figure 8:**
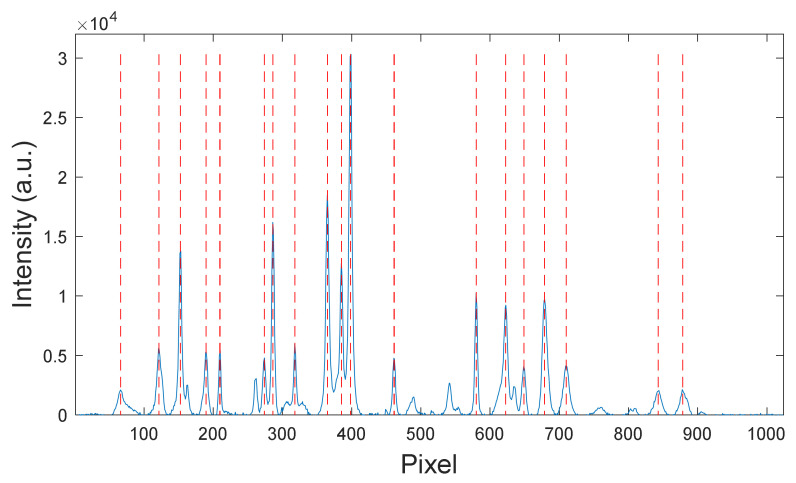
Peak-finding results for acetaminophen.

The Raman spectrometer used in this study used a semiconductor laser with a wavelength of 784.5 nm and an output power of 500 mW. After the spectrometer calibration system was built and the upper computer serial port was connected, the spectra of the substance were collected. The text file containing the spectral data captured a total of 2048 pixel positions for the high- and low-wavenumber bands (i.e., 1024 pixel positions for each band). First, the spectral data of the 1024 pixels in the low-wavenumber band were analyzed. A baseline correction algorithm was used to remove the baseline of the measured acetaminophen spectrum, which eliminated its influence on the peak-finding process [33]. The spectral peaks were found using the Gaussian fitting method, and the serial numbers of the corresponding pixels were obtained [34]. The results of the peak algorithm for acetaminophen are shown in Figure 8. The wavenumbers corresponding to the spectral peaks were determined according to the standard wavenumber values, as shown in Table 2.

The data from Table 2 were fit with a cubic polynomial, and the best-fit equation was derived using the polyfit command in the MATLAB platform, resulting in

(4) $$k\;\approx \;78.2+2.1\;\cdot \;i-\;4.1\;\times \;10^{-4}\;\cdot \;i^{2}-\;3.6\;\times \;10^{-8}\;\cdot \;i^{3}.$$

This formula represents the relationship between the pixel *i* and the wavenumber *k* in the low-wavenumber band of the spectrometer.

To calibrate the high-wavenumber band of the spectrometer, a halogen light source with a continuous spectral output curve in the 360–2500 nm range was connected to an OFC to generate comb-like spectral signals with equal wavenumber intervals. The wavenumber of each spectral peak of the OFC was calculated according to the best-fit equation for the low-wavenumber band (Equation (4)), and then was substituted into Equation (1) to calculate the wavenumber interval $\;{\mathrm{OFC}}_{\mathrm{WNspace}}\;$≈ 20.8${\;\mathrm{cm}}^{-1}$.

The peak-finding algorithm identified the pixel position ${\mathrm{ACN}}_{\mathrm{pixel}}$ of the acetonitrile 2253 ${\;\mathrm{cm}}^{-1}$ peak at 460.7 pixel. The peak-finding algorithm also discovered that the nearest OFC peak pixel position was ${\mathrm{OFC}}_{\mathrm{nearest}-\mathrm{Pixel}}$ = 463.3 pixel, the peak number was *n* = 44, and the next adjacent OFC pixel position was ${\mathrm{OFC}}_{\mathrm{second}-\mathrm{Pixel}}$ = 452. The total number of OFC peaks was *N* = 78. The wavenumber of the OFC peak was calculated using

(5) $${\mathrm{OFC}}_{\mathrm{nearest}-\mathrm{Pixel}}=2253+\frac{\left(463.3\;-\;460.7\right)}{\left(463.3\;-\;452.6\right)}\;\times \;20.8=2258.1\;{\mathrm{cm}}^{-1}.$$

Next, the wavenumber of spectral peak *i* was calculated according to the spectral peak serial number and the wavenumber of the OFC spectral peak interval:

(6) $${\mathrm{OFC}}_{i-\mathrm{WN}}=2258.1\;-\left(44\;-{\;\mathrm{OFC}}_{i-\mathrm{Index}}\right)\;\times \;20.8=1342.9+{\;\mathrm{OFC}}_{i-\mathrm{Index}}\;\times \;20.8.$$

The wavenumber of each spectral peak of the OFC was fitted using the least-squares cubic polynomial and the best-fit calibration formula of the mapping relationship between the wavenumber and the pixel in the high-wavenumber band according to

(7) $$k\;\approx \;1.2\;\times {\;10}^{3}+2.6\;\cdot \;i-\;6.9\;\times \;10^{-4}\;\cdot \;i^{2}-\;8.2\;\times {\;10}^{-9}\;\cdot \;i^{3}.$$

After the wavelength calibration was complete, it was necessary to calibrate the relative intensities of the two spectra in the high- and low-wavenumber bands. By comparing the blackbody radiation curve of a standard light source with the spectrum measured by the spectrometer under the same conditions, the response curve of the spectrometer can be obtained. In reality, no ideal blackbody light source is available. Because the spectrum of a halogen–tungsten lamp is more similar to that of a blackbody than that of other light sources, a halogen–tungsten lamp is often used for intensity calibration [35]. In this study, a halogen–tungsten lamp that continuously generated a smooth spectral curve in the 360–2500 nm wavelength range was used. The intensity calibration curve was obtained by comparing the spectrometer output with the output of the halogen tungsten source [36].

**Figure 9:**
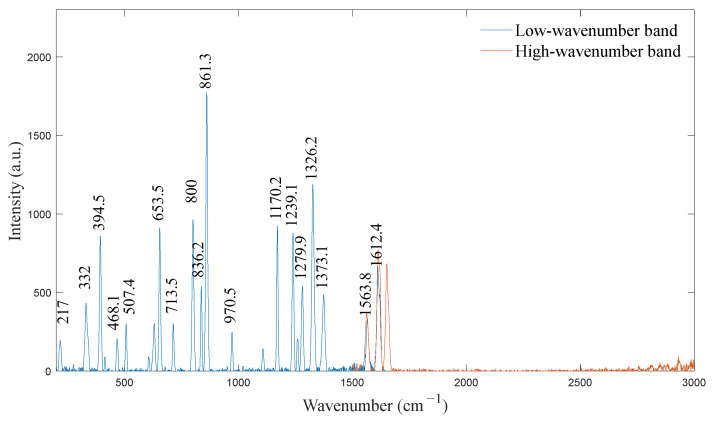
Splicing of the high- and low-wavenumber bands was calibrated using acetaminophen.

Finally, the spectra of the high- and low-wavenumber bands calibrated using acetaminophen were spliced, as shown in Figure 9. The calibration was thus complete.

### 4.2. Experimental Verification

**Figure 10:**
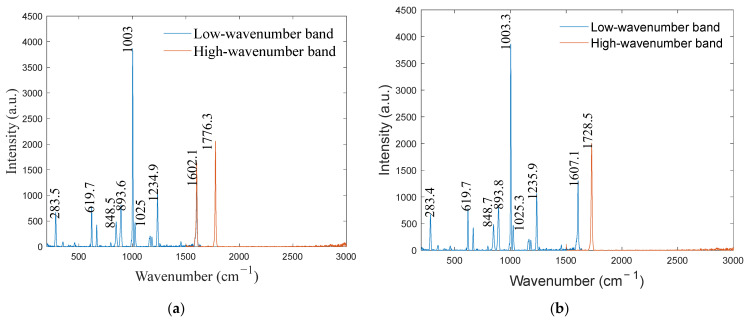
Benzoyl peroxide spectrum: (**a**) optical frequency comb calibration and (**b**) mercury–argon lamp calibration.

**Figure 11:**
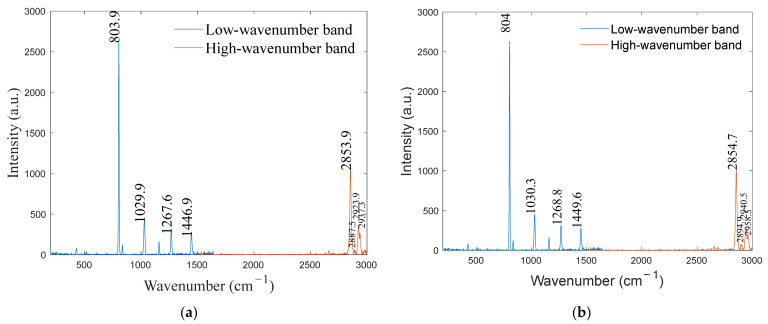
Cyclohexane spectrum: (**a**) optical frequency comb calibration and (**b**) mercury–argon lamp calibration.

**Figure 12:**
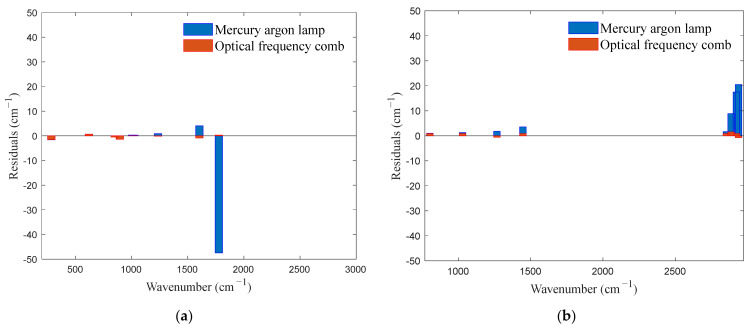
Distribution of the residuals of the calibration curves for (**a**) benzoyl peroxide and (**b**) cyclohexane.

To verify the calibration accuracy, the calibrated spectrometer was used to detect benzoyl peroxide and cyclohexane. The detection results were compared with the standard Raman spectra of the substances, and the calibration error was analyzed. Additionally, the calibration results of the proposed method were compared with those of traditional mercury–argon lamps, and the calibration accuracy was analyzed. The spectra of the benzoyl peroxide and cyclohexane are shown in Figure 10 and Figure 11, respectively, and the calibrated spectral peak wavenumbers of the benzoyl peroxide and cyclohexane are shown in Table 3 and Table 4, respectively. The distributions of the residuals of the calibration curves for the two substances are shown in Figure 12.

The calibration results for the positions of the standard spectral peaks indicated that the calibration performed according to the mercury–argon lamp exhibited significant errors. The maximum absolute error for the benzoyl peroxide detection results reached 47.5 ${\mathrm{cm}}^{-1}$, and the average absolute errors for benzoyl peroxide and cyclohexane were 6.3 ${\mathrm{cm}}^{-1}$ and 7.0 ${\mathrm{cm}}^{-1}$, respectively. However, the maximum absolute error for both benzoyl peroxide and cyclohexane, as determined by the OFC calibration, was 1.5 ${\;\mathrm{cm}}^{-1}$, and the average absolute errors for benzoyl peroxide and cyclohexane were 0.6 ${\mathrm{cm}}^{-1}$ and 0.9 ${\mathrm{cm}}^{-1}$, respectively. Figure 12 shows that the distribution of the residual errors of the OFC calibration was relatively uniform, with values no greater than 1.5 ${\mathrm{cm}}^{-1}$, and that the error bandwidth was substantially smaller than that of mercury–argon lamp calibration. These results demonstrated that the OFC significantly improved the calibration accuracy.

However, this experiment has its limitations. The sample size is insufficient, and the potential issues associated with this method will be identified and addressed through the analysis of a more extensive range of diverse substances in future investigations. Additionally, using other substances to calibrate the peak positions of the spectral comb causes very minor secondary errors. Exploring a more precise peak position-finding method for the OFC could further alleviate this error.

## 5. Conclusions

In this study, a wavenumber calibration method based on an OFC was proposed for a Raman spectrometer with dual channels and a wide spectral range. This method was convenient to operate, saved time, and achieved high accuracy. Acetaminophen, which has a large number of uniformly distributed spectral peaks, was selected to calibrate the low-wavenumber band. For the high-wavenumber band, acetonitrile and an OFC were combined to generate dense and uniform comb-like spectral signals at equal wavenumber intervals. The high- and low-wavenumber bands were calibrated via polynomial fitting, which addressed the problem of sparsely distributed characteristic spectral peaks and substantially improved the calibration accuracy for the high-wavenumber band of the dual-channel spectrometer. After the calibration was complete, the spectral data for benzoyl peroxide and cyclohexane were obtained, and the calibration results were verified experimentally. The experiments showed that the absolute error of the calibration results of the proposed method was not more than 1.5 ${\mathrm{cm}}^{-1}$, and that the average absolute error for each substance was significantly smaller than that of a mercury–argon lamp. These results demonstrated that the proposed method was simple, saved time, and improved calibration accuracy, which can offer helpful guidance for the calibration requirements of dual-channel Raman spectrometers. However, this experiment does have its limitations, notably the insufficient sample size and the possibility of secondary deviations. Moving forward, additional experiments will be conducted to scrutinize potential issues with this method. Moreover, efforts will be made to broaden the OFC across the entire spectral range, aiming for a more simplified approach.

## Figures and Tables

**Table 1:** Technical parameters of the spectrometer.

Parameter	Value
Laser wavelength	784.5 nm
Spectral range	200~3000 cm^−1^
Resolution ratio	2.6 cm^−1^
Pixel size	14 × 14 μm
CCD pixel	1024 × 64 pixels

**Table 2:** Comparison between the calibrated acetaminophen wavenumbers obtained in this study and the standard acetaminophen wavenumbers.

Standard Wavenumber $({\mathrm{cm}}^{-1}$)	Pixel Position (pixel)	Calibration Wavenumber $({\mathrm{cm}}^{-1}$)	Deviation $({\mathrm{cm}}^{-1}$)
217	66.1	217.0	0
332	121.6	332.0	0
394	152.6	394.5	0.5
468	189.8	468.1	0.1
507	209.7	507.4	0.4
654	286.4	653.5	−0.5
713	318.1	713.5	0.5
800	365.1	800.0	0
837	385.3	836.2	−0.8
861	398.5	861.3	0.3
971	461.4	970.5	−0.5
1170	580.1	1170.2	0.2
1239	622.7	1239.1	0.1
1280	648.9	1279.9	−0.1
1326	678.8	1326.2	0.2
1373	710.2	1373.1	0.1
1564	843.1	1563.8	−0.2
1612	878.4	1612.4	0.4

**Table 3:** Results and errors of spectral peak wavenumbers of benzoyl peroxide after calibration via a mercury–argon lamp and the OFC.

Standard Wavenumber $({\mathrm{cm}}^{-1}$)	Mercury–Argon Lamp Calibration $({\mathrm{cm}}^{-1}$)	Deviation $({\mathrm{cm}}^{-1}$)	Optical Frequency Comb Calibration $({\mathrm{cm}}^{-1}$)	Deviation $({\mathrm{cm}}^{-1}$)
285	283.4	−1.6	283.5	−1.5
619	619.7	0.7	619.7	0.7
849	848.7	−0.3	848.5	−0.5
895	893.8	−1.2	893.6	−1.4
1003	1003.3	0.3	1003.0	0
1025	1025.3	0.3	1025.0	0
1235	1235.9	0.9	1234.9	−0.1
1603	1607.1	4.1	1602.1	−0.9
1776	1728.5	−47.5	1776.3	0.3

**Table 4:** Results and errors of spectral peak wavenumbers of cyclohexane detection after calibration via a mercury–argon lamp and the OFC.

Standard Wavenumber $({cm}^{-1}$)	Mercury–Argon Lamp Calibration $({cm}^{-1}$)	Deviation $({cm}^{-1}$)	Optical Frequency Comb Calibration $({cm}^{-1}$)	Deviation $({cm}^{-1}$)
803	804.0	1.0	803.9	0.9
1029	1030.3	1.3	1029.9	0.9
1267	1268.8	1.8	1267.6	0.6
1446	1449.6	3.6	1446.9	0.9
2853	2854.7	1.7	2853.9	0.9
2886	2894.9	8.9	2887.5	1.5
2923	2940.5	17.5	2923.9	0.9
2938	2958.5	20.5	2937.3	−0.7

## Data Availability

Data are contained within the article.
